# Imaging of T-cells and their responses during anti-cancer immunotherapy

**DOI:** 10.7150/thno.37924

**Published:** 2019-10-16

**Authors:** Massis Krekorian, Gilbert O. Fruhwirth, Mangala Srinivas, Carl G. Figdor, Sandra Heskamp, Timothy H. Witney, Erik H.J.G. Aarntzen

**Affiliations:** 1 Department of Tumor Immunology, Radboud Institute for Molecular Life Sciences, Radboud university medical center, Nijmegen, The Netherlands; 2 Department of Radiology and Nuclear Medicine, Radboud university medical center, Nijmegen, The Netherlands; 3 Department of Imaging Chemistry and Biology, School of Biomedical Engineering and Imaging Sciences, Kings' College London, London, United Kingdom

**Keywords:** Immunotherapy, cell-based therapy, *in vivo* imaging, T-cells, positron emission tomography.

## Abstract

Immunotherapy has proven to be an effective approach in a growing number of cancers. Despite durable clinical responses achieved with antibodies targeting immune checkpoint molecules, many patients do not respond.

The common denominator for immunotherapies that have successfully been introduced in the clinic is their potential to induce or enhance infiltration of cytotoxic T-cells into the tumour. However, in clinical research the molecules, cells and processes involved in effective responses during immunotherapy remain largely obscure. Therefore, *in vivo* imaging technologies that interrogate T-cell responses in patients represent a powerful tool to boost further development of immunotherapy.

This review comprises a comprehensive analysis of the *in vivo* imaging technologies that allow the characterisation of T-cell responses induced by anti-cancer immunotherapy, with emphasis on technologies that are clinically available or have high translational potential. Throughout we discuss their respective strengths and weaknesses, providing arguments for selecting the optimal imaging options for future research and patient management.

## Introduction

Immunotherapy has shown promising outcomes in multiple cancer types 1. In the past years, the Food and Drug Administration (FDA) and European Medicines Agency (EMA) have approved several monoclonal antibody-based therapies targeting the immune checkpoint molecule programmed cell death receptor 1 (PD-1/CD279) or its ligand 1 (PD-L1/CD274) and cytotoxic T lymphocyte-associated antigen 4 (CTLA-4/CD152), based on large randomised clinical trials in *e.g.* melanoma 1-3, non-small cell lung cancer 4, 5 and renal cell carcinoma 6. Blocking these inhibitory pathways involved in peripheral tolerance effectively unleashes endogenous anti-cancer T-cell responses 7, 8. Alternatively, cell-based approaches such as chimeric antigen receptor (CAR) T-cells, which are T-cells endowed with fusion proteins that include both antigen-recognition moieties and T-cell signalling domains, have demonstrated remarkable responses 9. The antigen-recognition domain of these therapeutic cells is mostly derived from a monoclonal antibody targeting a tumour antigen, e.g. CD19 in the context of lymphoma. Infrastructures for centralised manufacturing and recent clinical trials have accelerated approval of the first CAR T-cell products for B-cell lymphoma and B-cell acute lymphoblastic leukaemia 10-12. These initial clinical successes of both immunotherapeutic approaches have resulted in recent rush for more effective (combination) treatments 13, 14. Despite the beneficial effects of immune checkpoint inhibitors and the emergence of cell-based therapies in clinical studies, their response rates are yet insufficient to implement these therapies in routine clinical practice 13, in addition to their high costs.

The main rationale for these immunotherapeutic approaches is to induce or enhance infiltration of cytotoxic T lymphocytes (CTL) into the tumour 15, 16. The signalling molecules and cellular components involved in these processes are conceptualised from preclinical mouse tumour models. However, mouse models in onco-immunological research are only moderately representative of humans since they have a different genetic and immunological background; not all human immune cell populations, metabolic enzymes and cytokines have a murine analogue, e.g. CXCL8 for the recruitment of neutrophils and T-cells 17, 18. Moreover, host-related factors such as age, sex and microbiome are increasingly being reported as relevant for the fitness of the immune system but differ markedly in mouse models as compared to the clinical context were elderly patients with co-morbidities and more heterogenous environments are treated 19, 20. Thus, many of the critical factors for successful expansion, infiltration of the tumour and execution of effector function of tumour-specific T-cells in patients remain unknown, until immunotherapeutic drugs are put to the test in clinical studies. The lack of biomarkers to assess ensuing immune responses in patients is one of the main hurdles in the further development of more effective anti-cancer immunotherapy.

Computed tomography (CT) measures the volume and enhancement patterns of tumours and is routinely incorporated in clinical trials for staging patients at baseline and monitor tumour responses during treatment. This information from CT, which is used for clinical decision-making and treatment development, however, does not inform on specific immunological pathways crucial for the efficacy of immunotherapy. Other clinical imaging modalities, such as positron emission tomography (PET), single photon emission tomography (SPECT) and magnetic resonance imaging (MRI) employ imaging tracers, which are specific for molecular targets, and have recently developed into clinically-applicable technologies. Therefore, novel *in vivo* imaging technologies to non-invasively assess immunotherapy-induced T-cell responses in cancer patients have the potential to become essential tools in the further development of immunotherapy 21, 22.

**Figure 1:**
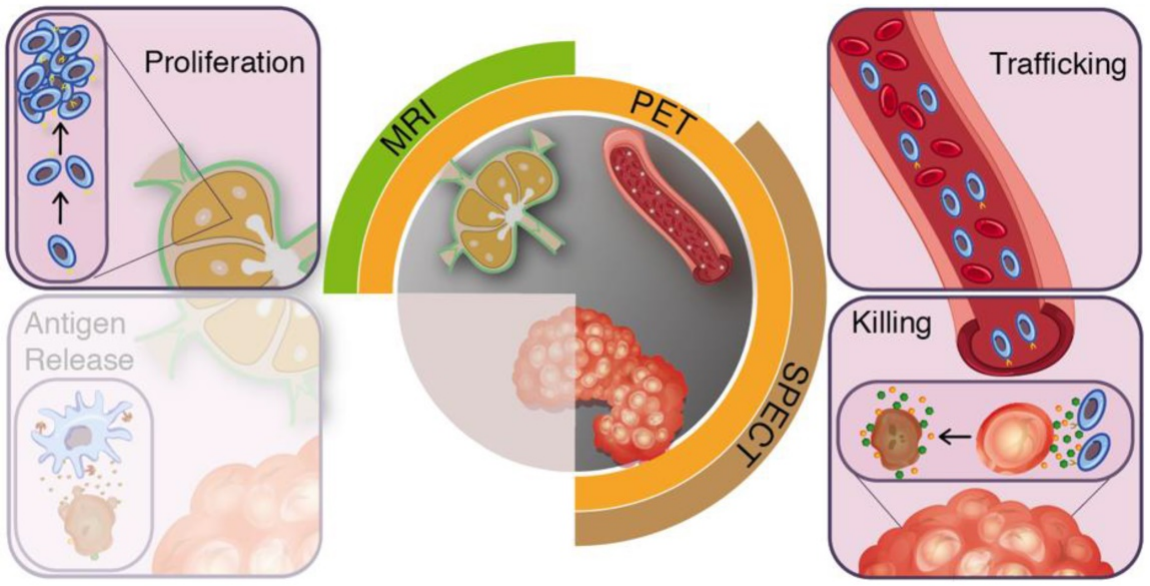
** Clinical imaging modalities and targets for T-cell imaging.** The effector arms of the anti-cancer immunity cycle involve T-cell proliferation, trafficking, and tumour-infiltration. Clinical applicable imaging modalities can target these steps during treatment induced T-cell responses.

**Figure 2:**
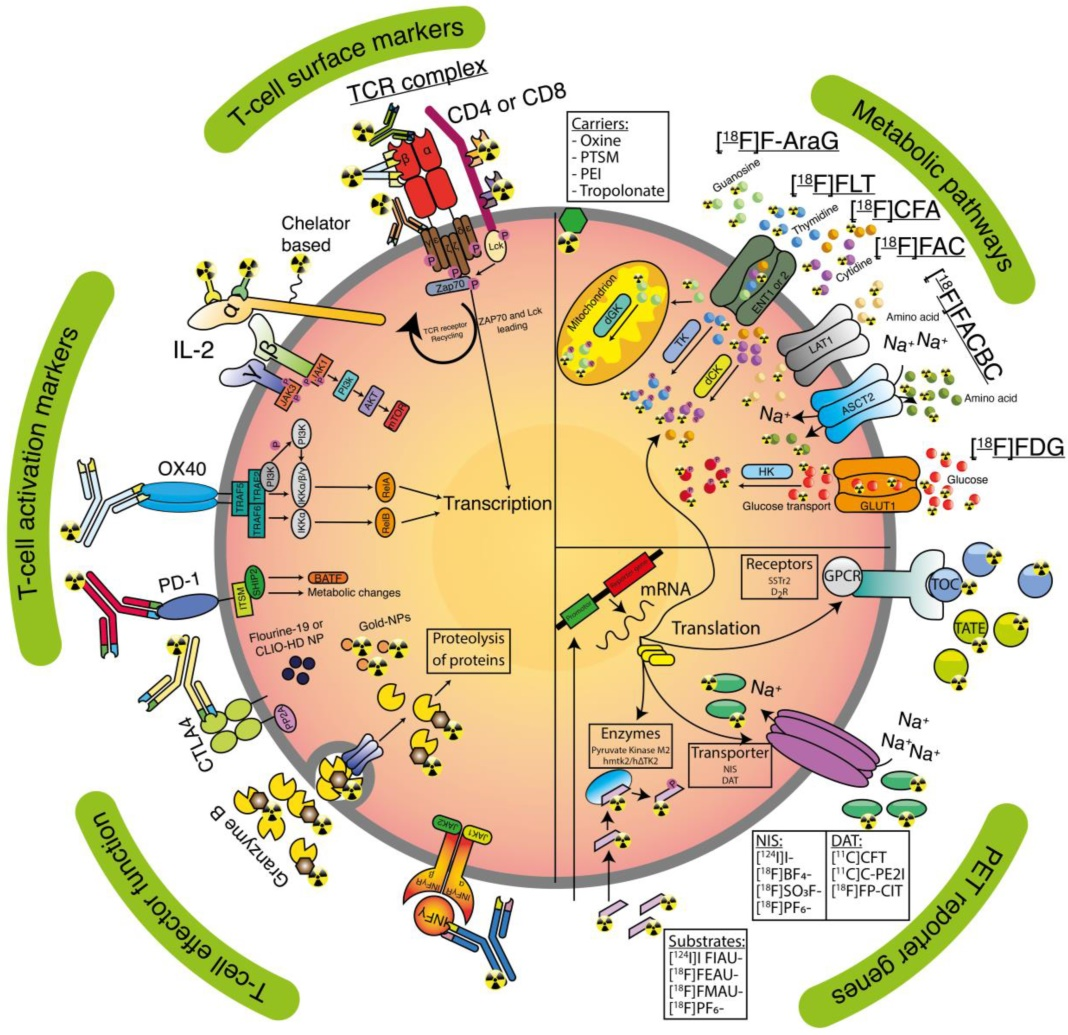
** Clinically applicable tracers and T-cell targeting imaging agents.** Receptors (activation markers and surface markers), lipophilic carriers, nanoparticles (NP), T-cells effector functions, metabolic targets, and PET reporter targets are represented as an overview for tracer targeting.

**Figure 3:**
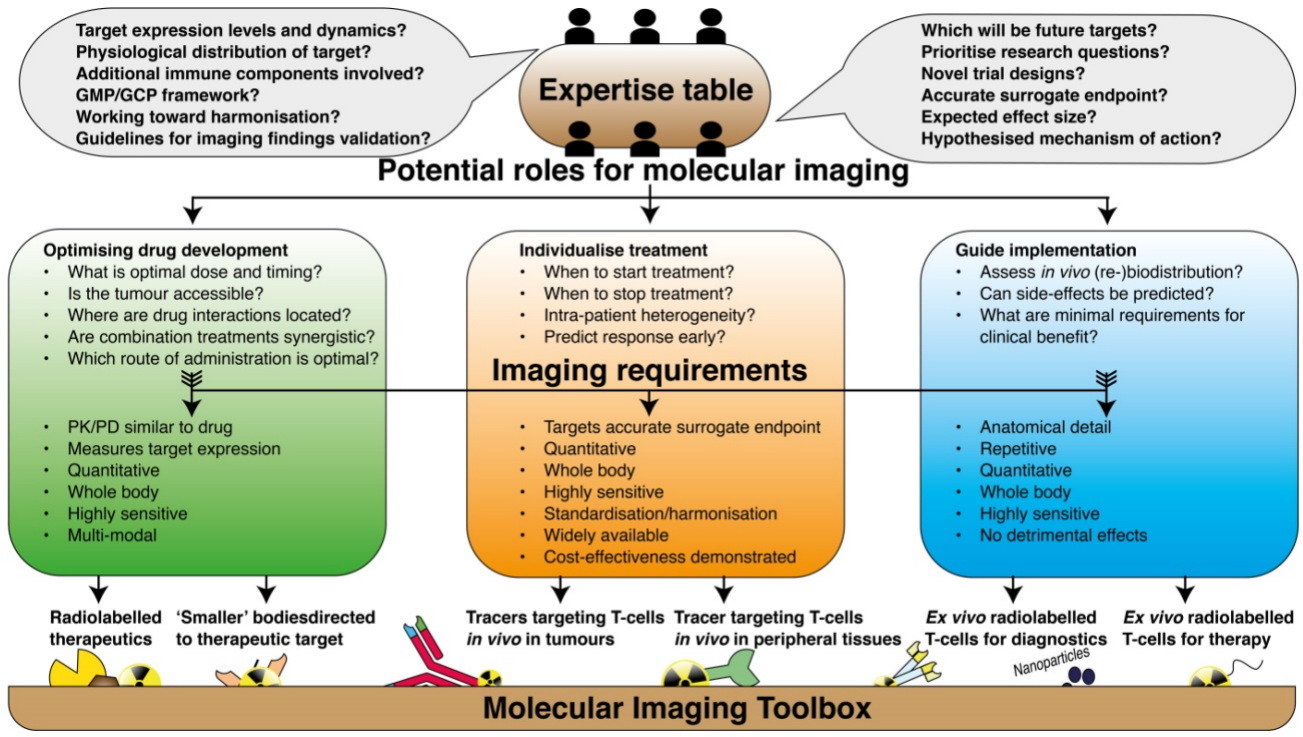
** Implementation of the molecular imaging toolbox in immune oncology.** Graphical illustration of the path towards implementation of current molecular imaging techniques in clinical research on immune therapy. In a collaborative approach; physicians, academic laboratories, pharmaceutical industries, scientific communities and regulatory bodies should acknowledge their complementary expertise and define the potential role that is envisioned for molecular imaging. The research questions should be defined in such way that it can directly be translated to decisions of which imaging tools are most suited for that particular purpose.

In the preclinical setting imaging technologies have already contributed greatly to our understanding of the conditions required for an effective anti-cancer immune response. Modalities such as intravital fluorescence microscopy and planar bioluminescence imaging yield vast amounts of valuable data as molecules and cells could be studied spatiotemporally at single cell resolution 23-26. Throughout this review, we will use the cancer-immunity cycle as a conceptual framework to guide our reasoning for medical imaging modalities, which provide tools to study T-cell responses *in vivo* in clinical studies, from their induction in the secondary lymphoid organs (SLO) *via* infiltration of tumours to activity measures in the tumour microenvironment (Figure 1 and 2). First, we will describe the cancer-immunity cycle with emphasis on targets and processes relevant for imaging purposes. Next, we will translate these immunological processes to open questions in current clinical immunotherapy research and matching imaging requirements (Figure 3). Lastly, we summarise available imaging technologies for *in vivo* evaluation of T-cells during immunotherapy.

## The cancer-immunity cycle

In recent years, the cancer-immunity cycle has been introduced as a concept to describe the necessary steps for effective anti-cancer immune responses 15. Endogenous anti-cancer immune responses initiate at the tumour where activated tissue resident antigen presenting cells (APC) recognize antigenic tumour fragments and migrate to SLO. Upon antigen-specific recognition and activation, T-cell populations expand and egress from the SLO. On site, T-cells infiltrate the tumour microenvironment and execute their cytotoxic effector functions. In *effective* anti-cancer immune responses, tumour destruction and subsequent inflammation results in the induction of new waves of effector cells, thereby continuing the cancer-immunity cycle 15, 16.

SLOs, in particular tumour draining lymph nodes (LNs, 27) and tertiary lymphoid structures 28, are hubs where activated APC and soluble tumour-derived fragments entering from the afferent lymph vessels meet naïve T-cells, which traffic in and out, screening for antigens 24, 29, 30. Upon antigen-recognition and adequate co-stimulation, naïve T-cells become activated, which is characterised by increased expression of several cell surface markers, which can be targeted for imaging (Table 1 and Figure 2). For example, the alpha chain of the trimeric IL-2 receptor (IL-2Ra/CD25) and cell-surface marker tumour necrosis factor receptor superfamily member 4 (TNFRSF4/OX40/CD134) 8. Furthermore, T-cells upregulate their expression of checkpoint molecules, e.g. CTLA-4 and PD-1, which allows to tone down their state of activation to preserve the delicate balance between effective cytotoxicity and host tissue integrity. As interaction *via* these receptors inhibits T-cell function, these are utilised in immune checkpoint inhibition 31. Activated T-cells clonally expand before they egress from the SLO and undergo metabolic reprogramming that allows them to survive and function in peripheral tissues 32, 33. Metabolic pathways that are upregulated in activated lymphocytes include glycolysis (in adjunct to oxidative phosphorylation in quiescent states and glutaminolysis 34), as well as nucleic acid metabolism 35 (Table 2 and Figure 2). Both early activation markers and metabolic reprogramming are indicators of successful immune induction.

Upon *in vivo* expansion, antigen specific T-cells egress from the LNs *via* the lymphatics or blood vessels, migrating to inflamed sites 36-38. Combined expression of specialised receptors on T-cells influences their preferential homing to peripheral tissues. For example, chemokine C-X-C receptor 3 (CXCR3), a receptor for the chemokines CXCL9 and CXCL10 expressed on endothelial cell surfaces, mediates T-cell extravasation and engraftment in the tumour 39-42. Tissue-resident memory T-cells release interferon-γ (INFγ), which led to upregulation of vascular cell adhesion molecule 1 (VCAM-1) on endothelial cells and enhanced recruitment of circulating T-cells to tumours 43. The longevity and distribution of T-cells across tumour sites and other peripheral tissues dictates the often delicate balance between treatment efficacy and immune-related adverse events. These two factors are important parameters to be assessed by non-invasive means for both *ex vivo* expanded and manipulated T-cell populations in adoptive cell transfer (ACT) as well as *in vivo* stimulated T-cells in therapy setting.

Upon arrival to the tumour, new challenges arise for T-cells to execute their effector functions. The tumour microenvironment is harsh to T-cells, which have to interact with a stiff and dense extracellular matrix 44, 45. Suppressive signals from various cells types including myeloid-derived suppressor cells and regulatory T-cells, e.g. PD-L1 and tumour growth factor beta (TGF-β) restrain T-cell infiltration and full development of their effector functions 1, 15, 46. In contrast, immune submissive tumour microenvironments permitted T-cell infiltration down to the core of the tumour, which is followed by the release of several effector molecules, *e.g.* granzyme B, and INFγ, resulting in cancer cell death 47, 48. The extent to which the tumour microenvironment is infiltrated by T-cells and execution of cytotoxic functions occurs classifies cancer in three main categories: inflamed (tumour core infiltrated by immune cells), excluded (the tumour is surrounded but not infiltrated by immune cells), and ignored (no T-cells present at tumour) 47, 48. Importantly, this categorization has high prognostic impact 49, 50, and is most likely predictive in the context of immunotherapy 51.

### *In vivo* imaging in the cancer-immunity cycle

Imaging early steps in T-cell activation and expansion provides a tool to check for correct delivery or *in situ* immune induction. Therapies that engage at these early steps in the cancer immunity cycle, with vaccines, adjuvants or local treatment, result in tissue-damage associated inflammation and antigen release. Imaging early T-cell responses in LNs should be considered *in situ*ations where it is crucial to detect developing adverse events at the earliest possible stage, when immune suppressive interventions can still be effective. Although upregulated metabolic pathways or cell surface activation markers are not per se linked to the actual increase in number of T-cells, the magnitude of imaging signal increase gives a hint on successful immune induction.

Upon intravenous injection, endogenously stimulated or adoptively transferred T-cells distribute systemically within hours to days, with prolonged retention found in spleen, bone marrow, and LN, and generally in rather low amounts in tumours. Given their potency, these rather small absolute numbers of cells per tumour volume can still induce profound responses 52. From an imaging perspective, tracking small number of cells implies a need for highly sensitive and whole-body imaging techniques; SPECT, PET and MR in preclinical studies, with PET mostly used for clinical purposes. Furthermore, repetitive imaging over long time points (weeks, months) is required to track the fate of injected cells which are marked with labels that do not decay and dilute upon cell division 53. Finally, in particular for tracking therapeutic cells intended to persist *in vivo*, it is critical to avoid the use of labels that can have detrimental effects on cell viability or function 54, 55. ^89^Zr-labelled cells with up to 0.5 Bq/cell showed no reduced viability, while 9.62 mBq/cell for ^111^In-labelled cells was without toxicity.

As responses to immunotherapy have shown a variety of patterns in clinical studies, which includes an initial increase in tumour size before regression occurs, termed pseudoprogression 56, time-to-response is uncertain and tumour shrinkage is a late endpoint resulting from complex interactions. These complex interactions are due to influx of T-cells during immune activation and eventual decrease in tumour size as a result of tumour cell killing 57. Quantification of the on-going T-cell infiltration of the tumour and local anti-tumour effector functions should allow clinicians to define thresholds for 'success' of immunotherapy at earlier stages during treatment. High specificity and quantitative imaging tools are required to guide drug development and tailor treatment to patient individual response characteristics. In this respect, monoclonal antibodies and in particular antibody fragments are favourite targeting moieties given their excellent specificity and good tissue penetration. These targeting agents have notably different biological half-lives 58, i.e. full antibody half-life in the circulation of ^89^Zr-labelled nivolumab is 26 days in patients when administered at 3 mg/kg every 2 weeks, as compared to 1-2 hrs for ^18^F-labelled adnectin BMS-986192 59, which requires consideration upon tracer generation. The biological half-life of these agents must match the dynamics of the biological process under study, and the radioisotope half-life should facilitate this. For example, tumour-infiltration and persistence by T-cells or induction of T-cell proliferation generally develops over days, which are slower than receptor expression per cell, which changes rapidly upon stimulation. Moreover, sufficient contrast is required to detect small numbers of T-cells, which can be achieved using tracers with high specific molar activities, await background clearance or by saturating sink organs using unlabelled tracer 60.

## Cells surface markers for *in vivo* imaging of T-cell activation

Immune therapeutic strategies can engage at the LN stage in the cancer-immunity cycle, such as dendritic cell DC-based therapy 61-63, which aim to deliver fully activated DCs loaded with tumour antigens to induce tumour-specific T-cell responses. Imaging techniques with sufficient anatomical detail, *e.g.* magnetic resonance (MR) and ultrasound, have been used to validate the correct delivery of these therapeutic vaccines, which is a critical step for the successful implementation of these biotherapeutics 64. However, in order to measure induced T-cell responses, molecular quantitative and sensitive imaging techniques such as SPECT and PET are better equipped to visualise subsequent T-cell activation, provided that the corresponding tracers are sufficiently specific (Table 1 and Figure 2). In this paragraph, we will address cell surface markers that are being explored for imaging small number of T-cells during activation.

The cytokine IL-2 has been used as a marker for activated T-cells, with increased expression of IL-2RA, in clinical studies using SPECT imaging. Metastatic melanoma patients on either ipilimumab or pembrolizumab therapy were injected with [^99m^Tc]Tc-HYNIC-IL-2 and prior to or after 12 weeks of treatment. In line with their previous studies in primary melanoma, [^99m^Tc]Tc-HYNIC-IL-2 accumulation was demonstrated in most melanoma metastases 65. Upon immunotherapy, some lesions showed increased uptake, whereas other lesions demonstrated decrease. The limited number of patients and availability of histological validation did not allow to draw conclusions on potential relation between the numbers of tumour-infiltrating lymphocytes and level of [^99m^Tc]Tc-HYNIC-IL-2 uptake. Furthermore, radiolabelled IL-2 is a bioactive cytokine, in this study one out of the five patients experienced grade 1 pruritis and grade 1 pain, both related to the infusion.

To overcome the poor sensitivity and spatial resolution of SPECT imaging in clinical setting, a ^18^F-labelled IL-2 tracer for PET imaging has been developed for PET imaging. An increase in N-(4-[^18^F]fluorobenzoyl)-interleukin-2 ([^18^F]FB-IL-2) uptake was shown when tumours were either irradiated or immunised, and further increase in uptake was observed when the treatments were combined, indicating a synergistic effect 66. To the contrary, a decrease in uptake was noted upon inhibition of cell migration using a CXCR4 antagonist 67. [^18^F]FB-IL-2 is currently being translated to the clinic (NCT02922283).

CXCR4 is a chemokine receptor expressed on different cell types of the hematopoietic system and involved in migration of cells of different hematopoietic lineages, *e.g.* bone marrow homing of stem cells and lymphocyte trafficking. Its natural ligand is CXCL12, also known as stromal-derived-factor-1 (SDF-1). Radiolabelled analogues of CXCR4 antagonists have been introduced in clinical imaging studies, predominantly in imaging multiple myeloma, and more recently in cardiovascular and infectious diseases 68, 69. To date, no studies on radiolabelled CXCR4 ligands in the context of immunotherapy have been reported.

PET can image T-cell activation status through targeting the activation marker OX40, which is upregulated on the surface of T-cells upon antigen-specific activation 70. Using a ^64^Cu-conjugated murine antibody specific for the OX40 receptor, PET imaging was used to measure T-cell activation localised in treated A20 lymphoma tumour, tumour-draining LNs as well as spleen, upon intratumoural injection of CpG oligodeoxynucleotide. At an early time point after *in situ* vaccination, this approach enabled prediction of anti-cancer immune responses. Given its role in anti-cancer immune responses, humanised OX40 agonist monoclonal antibodies are currently being introduced in early phase clinical trials for various cancer types (*e.g.* NCT02318394).

In summary, LNs are the hub for priming tumour-specific T-cells, where local antigens are transported to via DCs, *in situ* vaccination or therapeutic vaccines are presented to T-cells during early steps in the cancer-immunity cycle. Early markers of T-cell activation in lymph nodes provide indicators of successful immune induction. As a consequence of T-cell activation, T-cells start traversing the body and will quickly reach the tumour. However, expression of activation markers does not per se indicate cytotoxic effector functions, and this might restrict interpretation of imaging signal at the tumour sites. Moreover, the expression of this class of cell surface molecules is highly variable during immune responses, which hampers direct correlation of signal intensity with cell numbers.

### 3.1 Metabolic targets for imaging T-cell activation

Activated T-cells switch on additional metabolic programs and upregulate the influx of substrates, which is not seen in non-active cells in general. Targeting these metabolic pathways is therefore of interest as it enables the distinction between active and non-active T-cells (Table 2 and Figure 2). Several tracers have been developed that are substrates for key enzymes in the deoxyribonucleoside salvage pathway; deoxycytidine kinase (dCK) and deoxyguanosine kinase (dGK). For example, 1-(2′-deoxy-2′-[^18^F]fluoroarabinofuranosyl) cytosine ([^18^F]FAC) which has preferential *in vivo* distribution in lymphoid organs. In a onco-retrovirus tumour model, [^18^F]FAC enabled visualisation of immune activation in spleen and tumour-draining LNs. This uptake was 4-fold higher in CD62L^LOW^/CD44^HIGH^ effector CD8^+^ T-cells as compared to naïve T-cells, suggesting specificity for more mature population of T-cells 71.

^18^F-labelled clofarabine ([^18^F]CFA) is another nucleotide purine analogue metabolised *via* dCK. Previous translational studies showed a direct correlation between [^18^F]CFA accumulation and dCK expression in leukaemia cells, which could be blocked with a dCK inhibitor. In a first-in-human study, [^18^F]CFA PET/CT showed preferential accumulation in hematopoietic bone marrow and secondary lymphoid organs 72, 73. This tracer is currently being studied in metastatic melanoma patients undergoing TIM-3 targeted immunotherapy (NCT03409419). Furthermore, comparative biodistribution studies in healthy volunteers underscore the impact of probe affinity for other components of the targeted metabolic pathways, e.g. uptake transports and catabolic enzymes on the biodistribution. Higher bone marrow uptake was observed for L-[^18^F]FAC and L-[^18^F]FMAC than [^18^F]FAC, liver uptake was high for L-[^18^F]F-FMAC and L-[^18^F]F-FAC, whereas spleen and muscle uptake was highest for [^18^F]FAC 74.

**Figure 4:**
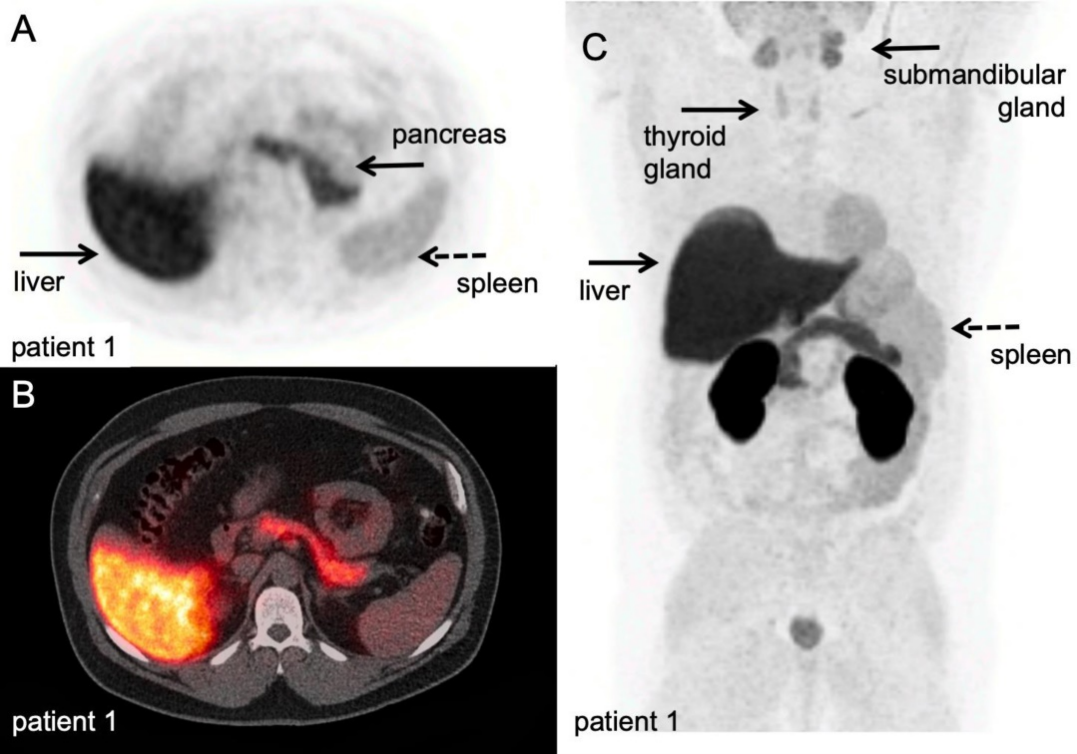
** [^18^F]F-AraG PET/CT scan.** A volunteer scanned at 60 minutes after IV injection of 189,07 MBq. A-B: transversal PET and fused PET/CT images of a volunteer; physiological [^18^F]F-AraG distribution *in vivo.* [^18^F]F-AraG exhibits hepatobiliary and renal clearance with highest uptake in associated organs at 60 minutes after IV tracer injection. C: Maximum intensity projection; relatively high uptake was observed in the myocardium, as seen in mice, and to lesser extend in the pancreas and spleen. Low background was observed in the thorax and lower abdomen. *With courtesy of CellSight Technologies Inc.*

Another PET tracer that has been developed to target T-cell specific metabolic pathways is 2′-deoxy-2′-[^18^F]fluoro-9-β-D-arabinofuranosylguanine ([^18^F]F-AraG). This tracer accumulates in activated T-cells predominantly *via* the dGK pathway and has previously been used a T-cell depleting drug in refractory/relapsed T-cell acute lymphoblastic leukaemia. In a murine model of acute graft-*versus*-host-disease (GVHD), PET imaging of [^18^F]F-AraG enabled the visualisation massive donor T-cell activation in SLO, preceding the onset of GVHD symptoms. Recent biodistribution study in healthy humans 75 (Figure 4), cleared the way for current clinical studies in cancer patients in the context of immunotherapy (NCT03311672, NCT03142204, NCT03007719).

The tracer trans-1-amino-3-[^18^F]fluorocyclo-butanecarboxylic acid ([^18^F]FACBC) is a synthetic amino acid that is taken up by activated immune cells 76, 77. [^18^F]FACBC uptake ratios of stimulated *versus* non-stimulated T-cells in a rat model rapidly increased in comparison to B-cells and macrophages. However, the ratio of [^14^C]C-FACBC uptake by T-cells compared to tumour cells was less than the uptake of 2-deoxy-2-[^18^F]fluoro-⫐-glucose ([^18^F]FDG) ratios, which might limit its utility for imaging activated T-cells in the tumour microenvironment.

**Figure 5:**
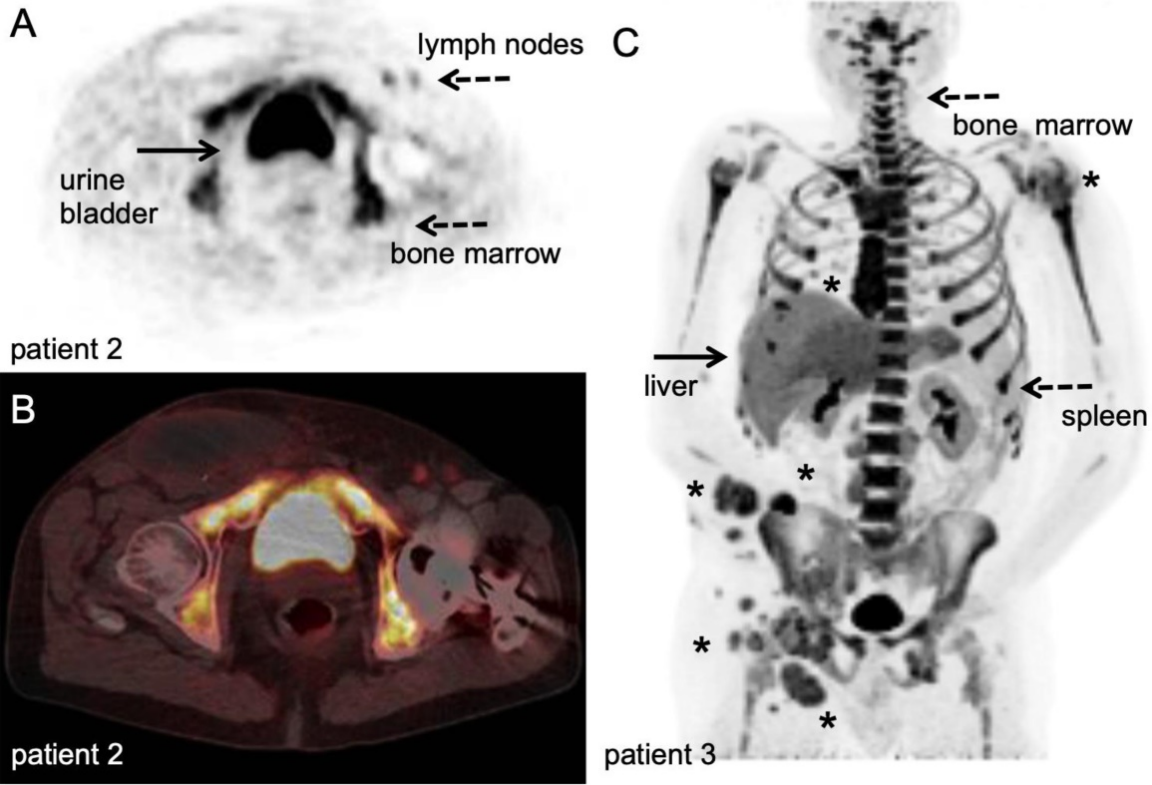
** [^18^F]FLT PET/CT example.** Stage III melanoma patient during adjuvant immune therapy, scanned at 60 minutes after IV injection of 306 MBq. A-B: transversal PET and fused PET/CT images; upon intranodal injection with antigen loaded dendritic cells, a clear [^18^F]FLT signal increase in the injected and subsequent draining lymph nodes in the left inguinal region (dashed arrow) was observed in correlation with antigen specific T-cell responses. Note the post-surgical changes in the right inguinal region after radical lymph node dissection *(*78*, unpublished results).* C: Maximum intensity projection;* in vivo* biodistribution of [^18^F]FLT in stage IV metastatic melanoma patient at baseline for start systemic treatment, scanned at 60 minutes after IV injection of 200 MBq at 3 days time interval. This patient has multiple lesions in lymph nodes, subcutaneous tissue and lung (asterisk). Note the high physiological activity in the haematopoietic system, uptake in the liver and to lesser extend in the spleen, and excretion *via* the kidneys. *With courtesy of B. van der Hiel.*

In a clinical study, PET imaging with 3'-deoxy-3'-[^18^F]fluorothymidine ([^18^F]FLT), a thymidine analogue targeting DNA-synthesis, has been used to monitor the kinetics and levels of antigen-specific T-cells in SLO after intranodal injection with antigen-loaded DC-based vaccines. Melanoma patients who received antigen-loaded DC showed an increase in [^18^F]FLT uptake in the injected LNs (Figure 5), whereas injection with saline or DC without antigen induced no [^18^F]FLT uptake in control LNs 78. In contrast to [^18^F]FDG, which measures rates of glycolysis in activated T-cells, the [^18^F]FLT signal was quantitatively correlated to the magnitude of antigen-specific T-cell proliferative responses measured in peripheral blood. In this setting, *ex vivo* labelling of DC-based vaccines for SPECT imaging confirmed that the observed signal in the LNs co-localised with the presence of antigen-loaded DCs and validated the concept that even small numbers (4.5 x 10^5^ cells) of APC can induce profound T-cell responses, as measured by a 2-3 fold signal increase and SUV_max_ up to 7.8, for up to 3 weeks.

Blockade of the coinhibitory molecule CTLA-4 has been shown to enhance T-cell responses. Patients with advanced melanoma treated the CTLA-4-blocking antibody tremelimumab, were analysed for changes in glycolysis using, [^18^F]FDG and DNA-synthesis using [^18^F]FLT. At a median of two months after start of treatment, no significant changes in size, [^18^F]FDG or [^18^F]FLT uptake in metastases were observed. However, the authors noted increased [^18^F]FLT uptake in the spleen, in the absence of changes in [^18^F]FDG uptake, as a consequence of immunotherapy 79. Unfortunately, the number of patients was too limited to correlate the changes in [^18^F]FLT uptake in the spleen to either tumour responses or adverse events.

**Figure 6:**
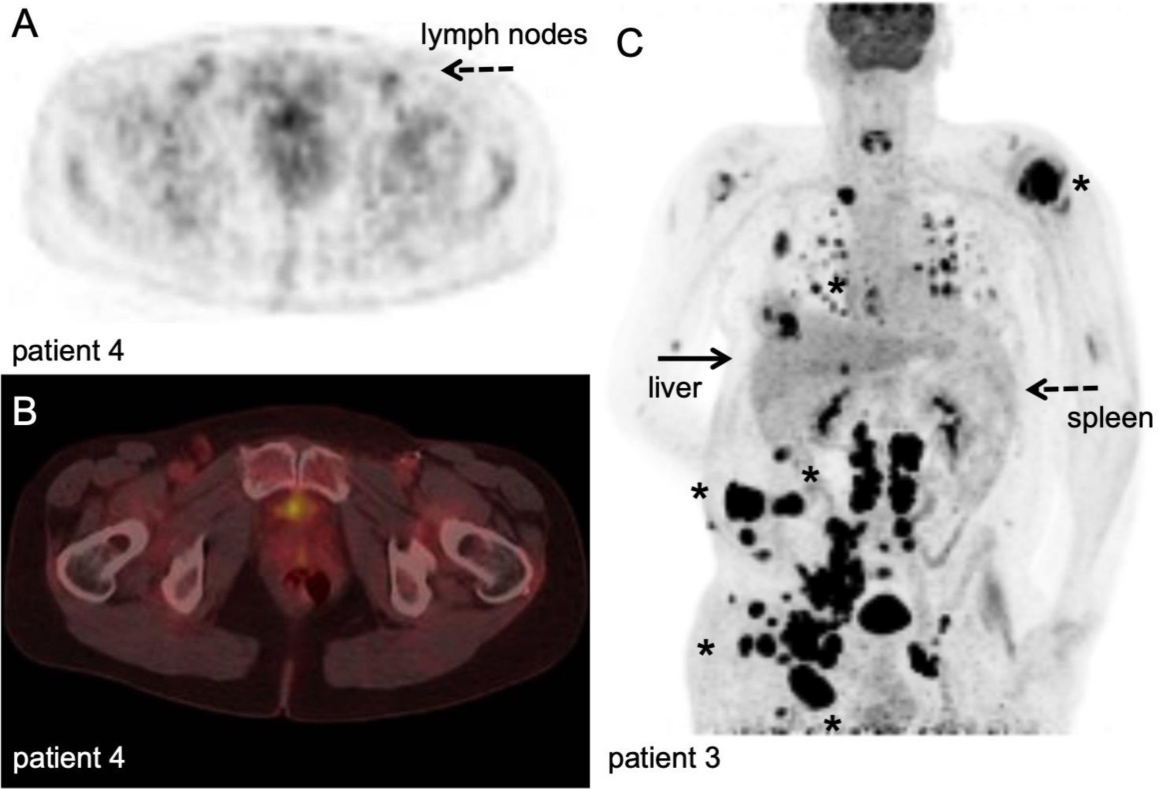
** [^18^F]FDG PET/CT example.** Stage III melanoma patient during adjuvant immune therapy, scanned at 60 minutes after IV injection of 207 MBq. A-B: transversal PET and fused PET/CT images; upon intranodal injection with antigen loaded dendritic cells, a faint signal increase in the injected and subsequent draining lymph nodes in the right inguinal region (dashed arrow) was observed which was not correlated to the magnitude of antigen specific T-cell responses. *(*78*, unpublished results).* C: Maximum intensity projection; *in vivo* biodistribution of [^18^F]FDG in the same stage IV metastatic melanoma patient as Figure 4 with multiple lesions in lymph nodes, subcutaneous tissue and lung (asterisk). Note the normal physiological activity as well as high uptake in the metastatic tumour lesions. *With courtesy of B. van der Hiel.*

Currently, [^18^F]FDG PET imaging to assess responses to immune checkpoint inhibition is under discussion 80. Literature suggests a role for [^18^F]FDG imaging to demonstrate early/hyper progression 81-83, but generally the high rates of glycolysis in tumour cells confounds interpretation of [^18^F]FDG signal at the tumour site. Although [^18^F]FDG PET imaging is used to assess immune-related adverse events 84 (Figure 6), its potential role to measure early therapy-induced metabolic switches from oxidative phosphorylation to glycolysis in SLO and haematopoietic system is only recently explored but warrants validation 85.

In summary, metabolic reprogramming licenses T-cells to develop into full effector phenotypes. As such, T-cell specific metabolic pathways are being investigated as imaging targets to assess ensuing immune responses at early time points. Signal increase in lymphoid compartments, e.g. bone marrow, spleen and SLO, can reliably be assessed as background signal is absent. However, as most tumours are metabolically plastic and exploit an array of metabolic pathways to fuel their energy demands, signal interpretation at the tumour sites will be challenging. A possible solution for imaging onsite in the tumor is to target specific T-cell surface receptors.

### 3.2 Cell surface markers for *in vivo* imaging of specific T-cell populations

**Figure 7:**
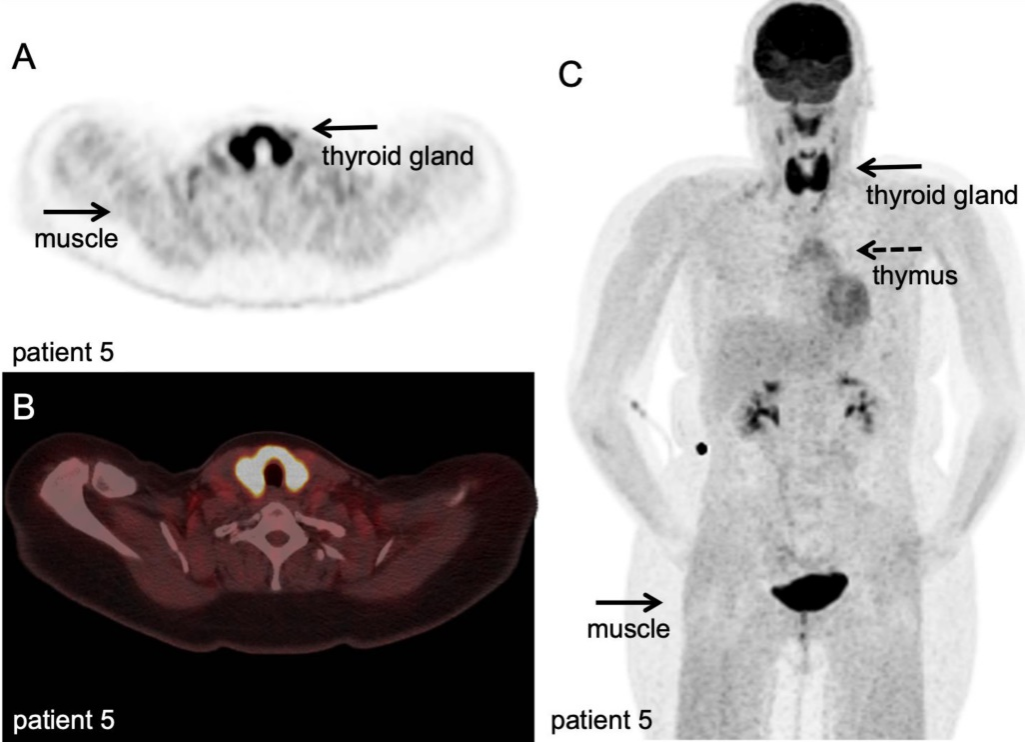
** [^18^F]FDG PET/CT example of immune related adverse events.** Hodgkin lymphoma patient under PD-1 targeting immunotherapy presenting with myalgia and muscle weakness, scanned at 60 minutes after IV injection of 298 MBq. A-B: transversal PET and fused PET/CT images; diffuse high [^18^F]FDG uptake in the thyroid gland, indicative of thyroiditis, and diffuse markedly increased [^18^F]FDG uptake in all muscles, matching the clinical symptoms of myositis. C: Maximum intensity projection; in addition to thyroiditis and myositis as immune-related adverse events, diffuse increased [^18^F]FDG uptake in the thymus. *(unpublished results)*

One approach to visualise T-cell responses is targeting the T-cell surface glycoprotein CD3 (Table 1 and Figure 2). This approach was employed during anti-CTLA-4 treatment in colon cancer xenograft models using a murine ^89^Zr-labelled anti-CD3 antibody ([^89^Zr]Zr-DFO-CD3) to quantify T-cell infiltration; interestingly high levels of infiltrations were found to precede tumour regression 86. Another cell surface marker, lymphocyte-activation gene 3 (LAG3), was targeted for localisation of tumour infiltrating T-cells in mice models bearing the human variant of the LAG3 (MC38/hLAG3). The full anti-LAG3 antibody labelled with [^89^Zr]Zr-DFO ([^89^Zr]Zr-REGN3767) is currently in phase 1 clinical trials for advanced malignancies, including lymphoma (NCT03005782). The researchers are investigating whether it is possible to use this method for predicting and monitoring therapy response of anti-LAG3 with and without anti-PD1 treatment. Full antibodies accumulate slowly in peripheral tissues, which imply imaging a day(s) after tracer administration in clinical studies with consequent negative implications on patient experience and cost to the health system. In contrast, antibody fragments reach their targets much faster and are rapidly cleared 87. In preclinical models, both ^89^Zr-labelled anti-CD4 and anti-CD8 cys-diabodies have been used to track respective T-cell populations. More recently, ^64^Cu-labelled anti-CD8 cys-diabody ([^64^Cu]Cu-169cDb) has been used for T-cell tracking in mice models. The choice for copper-64 instead of the more popular zirconium-89 was to reduce radiation exposure while the stability was comparable with [^89^Zr]Zr-malDFO-169cDb. The researchers were able to accurately visualise and quantify changes in response to immunotherapy with αPD1 and CpC. However, they point out that it is necessary to use multiple modalities for a full response assessment 88. In these studies, microdoses of the antibody fragments showed high contrast in PET images and minimal adverse effects on the T-cells, as demonstrated by a decrease in CD4 expression, inhibition of proliferation, and inhibition of INFγ production. Other studies demonstrated the feasibility of this approach to assess ensuing T-cell responses and intratumoural distribution during different immunotherapies, including ACT, anti-PD-L1 treatment, and combination treatment with anti-tumour necrosis factor receptor superfamily member 9 (4-1BB/anti-CD137) 87, 89, 90. Furthermore, engineered bispecific proteins targeting the 4-1BB molecule on T-cells and tumour stroma (FAP-4-1BBL) or tumour antigen (CD19-4-1BBL) have been developed to reduce hepatotoxicity and dependency on FcγR binding (hulgG1PGLALA). Labelling these proteins with radionuclides for PET/SPECT will further increase our capabilities to track T-cells for therapy 91. Clinical studies using engineered antibody fragments (minibodies) targeting CD8 are either completed (NCT03107663) or recruiting patients (NCT03802123, NCT03610061), providing showcases of the potential for *in vivo* imaging to optimise immunotherapy (Figure 7).

T-cell receptors (TCR) are an attractive group of imaging targets, particularly since constant membrane turnover results in internalisation and high accumulation of the tracer in the cells. Employing *in vivo* T-cell imaging or *ex vivo* T-cell labelling approaches, a ^64^Cu-labelled anti-chicken OVA-TCR antibody was efficiently internalised within 30 minutes, without impairing antigen recognition *via* the TCR receptor, viability or functionality 92. Others have taken a similar approach for *in vivo* T-cell imaging, tracking engineered human T-cells using ^89^Zr-labelled anti mouse TCR F(ab')2 fragment ([^89^Zr]Zr-Df-aTCRmu-F(ab')2), which is selective for the murine TCR beta domain of a transgenic TCR. In this approach, *ex vivo* labelling was optimized by exploiting the re-shuttling TCR to achieve higher specific activity. Intravenous injection with different numbers of transgenic T-cells, followed by injection of the F(ab')2 tracer, PET/CT imaging and *ex vivo* T-cell quantification in the tumour showed a good correlation between the total number of transgenic T-cells detected *ex vivo* and by PET imaging, which was independent of engraftment rates 93.

In summary, cell surface markers are interesting targets for imaging as they enable *in vivo* detection of specific cell populations. An array of tools is becoming available for this task including, full antibodies, cys-diabodies, minibodies, and F(ab')2 fragments. However, quantification of T-cell subpopulations via cell surface markers remains open for optimisation, as some of the targets may be expressed at different levels during the course of treatment and expression levels are generally unknown when scanning. Moreover, presence does not always imply a functionally effective phenotype, as the cytotoxicity is dependent on soluble factors (cytokines, availability of oxygen and metabolites) and cell-cell contact with stromal cells and regulatory immune cells. An interesting recent approach is the targeting of inhibitory pathways and other effector molecules for T-cells imaging, especially at tumour site.

### 3.3 Effector molecules for *in vivo* imaging of T-cells

Tumour cells upregulate PD-L1, which binds PD-1 on T-cells. PD-1/PD-L1 interaction does not directly result in T-cell death, but reduces the activity, proliferation and survival of T-cells 8 (Table 3 and Figure 2). Even though it is still early days in the development of monoclonal antibodies targeting immune checkpoint inhibitors, immunotherapy targeting the PD-1/PD-L1 axis has already had tremendous impact on the (immuno-)oncological research.

**Figure 8:**
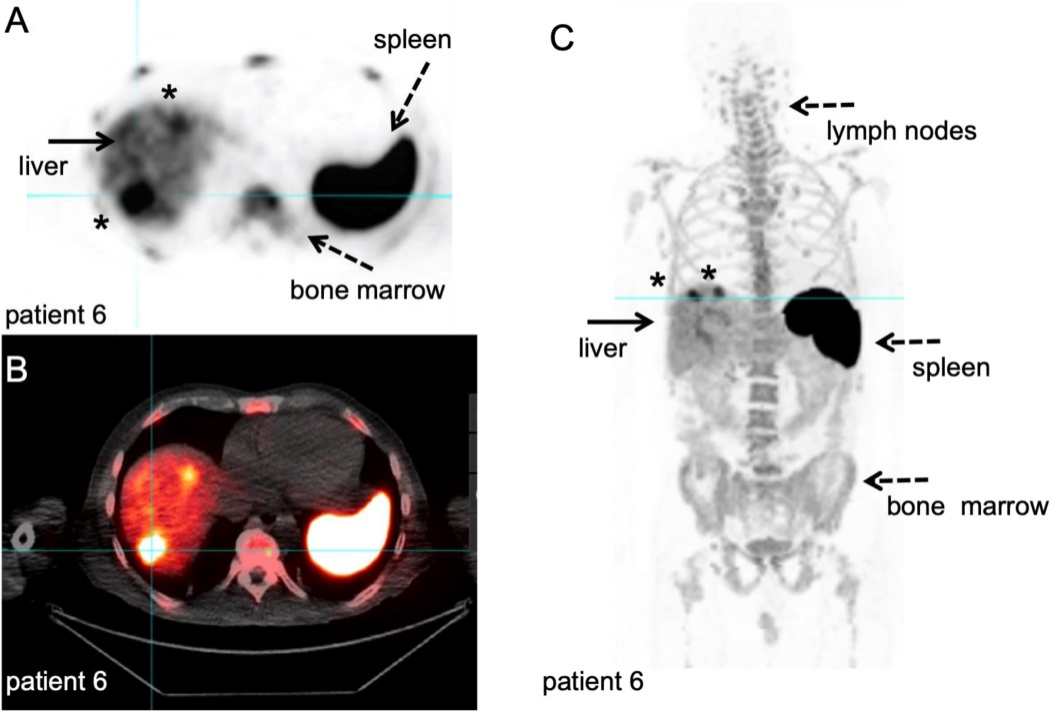
**^89^Zr-labelled anti-CD8 minibody PET/CT.** Patient with newly diagnosed Hepatocellular Carcinoma on immunotherapy for 12 weeks prior to IV injection of 111 MBq of ^89^Zr-Df-IAB22M2C. PET/CT images were acquired 24 hours after injection, for information on the molecule we refer to the publications that are currently in preparation. A-B: Axial ^89^Zr-Df-IAB22M2C PET and corresponding axial fused PET/CT images demonstrate two focal areas of ^89^Zr-Df- IAB22M2C uptake in the liver (filled arrow). The ^89^Zr-Df- IAB22M2C uptake in the lateral aspect of the right hepatic lobe corresponds to a 2.5 cm lesion (asterisk). The ^89^Zr-Df- IAB22M2C uptake in the anterior aspect of the left hepatic lobe (asterisk) is due to co-localization of ^89^Zr-Df- IAB22M2C to an occult hepatic metastasis. C: The coronal MIP PET image shows the intense uptake of ^89^Zr-Df-IAB22M2C in reference tissues with known areas of high CD8 TIL cells such as the lymph node (LN), spleen and bone marrow (dashed arrows). The two foci of ^89^Zr-Df-IAB22M2C focal uptake within the liver (filled arrow) is also clearly seen on this projection due to the relatively low hepatic background activity compared to CD8 rich tissues. This case demonstrates the value of ^89^Zr-Df-IAB22M2C PET scans to detect CD8 T-cells in the tumour microenvironment. *With courtesy of ImaginAb Inc.*

Clinical quantitative imaging of the *in vivo* biodistribution of these therapeutic antibodies can have direct repercussions on patient selection, optimisation of treatment schedules and design of novel combination therapies. The feasibility of this approach has been demonstrated in several preclinical studies 94, for example by ^64^Cu-labelled anti-PD-1 antibody 95. PET imaging over a period of 48h revealed tracer uptake in both lymphoid organs and tumour. Recently, a landmark first-in-human clinical study exploited ^89^Zr-labelled nivolumab in non-small cell lung cancer patients to assess PD-1 expression in the tumour prior to anti-PD-1 treatment 96. A correlation between PD-1 expressing lymphocytes in tumour biopsies, as determined by immunohistochemistry, and [^89^Zr]Zr-DFO-nivolumab uptake was observed. In this study, [^89^Zr]Zr-DFO-nivolumab uptake pre-treatment was higher in responding tumour lesions as compared to non-responding tumours, with a higher predictive score than gold-standard immunohistochemical markers (Figure 8). Along the same line, two studies on [^89^Zr]Zr-DFO-pembrolizumab imaging are currently open for locally advanced or metastatic melanoma or non-small cell lung cancer (NCT03065764, NCT02760225).

In order to get a broader understanding of the expression of CTLA-4, reduce cost and side effects of therapy, an monoclonal antibody targeting CTLA-4 (anti-CTLA-4) was conjugated with ^64^Cu-tracer ([^64^Cu]Cu-DOTA-anti-CTLA-4) 97. A mouse model of CT26 tumour bearing BALB/c mice was used to examine the expression of CTLA-4 on these tumours. They correlated the high expression detected to T-cell expression of CTLA-4 and not to the tumour cells. A phase 2 clinical trial with [^89^Zr]Zr-DFO-ipilimumab in metastatic melanoma patients is currently running (NCT03313323). Other effector molecules that are unequivocally associated with inflammatory anti-tumour immune responses are being explored as imaging targets (Table 3 and Figure 2).

The release of granzyme B a definite indicator of anti-tumour T-cell function, and has been targeted for PET imaging during immunotherapy 98, 99 with the aim to increase specificity in the detection of effective immune responses, rather than increased presence of T-cells. These studies demonstrate in several tumour models the high accuracy of PET imaging to predict response, with a quantitative correlation between signal intensity and magnitude of response. Using this approach, molecular imaging correctly predicted that sequential dose scheduling of PD-1 and CTLA-4 therapy was equally effective as concurrent administration; illustrating its potential to address relevant open clinical questions. To demonstrate translational potential, tumour biopsies from patients on checkpoint inhibitors were used to target granzyme B expression with [^68^Ga]Ga-NOTA-GZP. The probe was able to distinguish between responders and non-responders during therapy 98.

IFNγ is an attractive target for imaging immune responses due to its function in the T-cell signalling axis. Upon HER2/neu vaccination in spontaneous salivary and orthotopic neu+ mouse mammary tumour models, an ^89^Zr-labelled anti-IFNγ probe for PET imaging detected elevated cytokine levels, indicative of response to therapy. Specificity was confirmed in a model of induced T-cell exhaustion where CD8+ T cells infiltrate the tumour, but upregulate PD-1. In this model, IFNγ tracer uptake did not exceed isotype control, compatible with a lack of anti-tumour T-cell activity. As compared to imaging cell surface markers, which have background signals due to binding in secondary lymphoid tissues, targeting of soluble cytokines such as IFNγ may provide alternative tools and provide insight into the function of immune cells *in situ* 100.

In summary, direct *in vivo* assessment of the presence, numbers and functional status of specific immune cell populations during immunotherapy is feasible and to date the most advanced technology with respect to implementation in clinical research. First clinical studies employing tracers to this purpose provide proof-of-concept to translate concepts based on 2D immunohistochemistry of tumour tissue into 3D whole-body clinical imaging modalities.

## Imaging adoptively transferred T-cell therapy

Adoptively transferred T-cell-based immunotherapies differ from previously discussed molecular immunotherapy in that they are live cell products. Their longevity, distribution and potential re-distribution, as well as the overall survival of therapeutic T-cells across tumour sites and other tissues are critical parameters that need careful evaluation before the full potential of these approaches can be unrolled. For example, on-target off-tumour activities can lead to severe adverse effects 101, 102; CAR T-cells were associated with life-threatening side effects and fatalities during clinical trials 103, 104. However, clinical trials are still largely performed without knowledge about the *in vivo* distribution and fate of the administered therapeutic cells. Thus, at this point in time making it is impossible to adequately assess their safety and localisation and expansion at target site. Another area where imaging can play an important role is in GVHD. The main cause of this disease is thought to be failure for self-tolerance. Regulatory T-cells are mediators of self-tolerance and during chemotherapy these cells are diminished 22.

*In vivo* tracking of T-cell-based immunotherapy requires one of two strategies to label cells: either (i) 'direct cell labelling', whereby a contrast agent is directly loaded into the therapeutic cells (Table 4 and Figure 2); or (ii) 'indirect cell labelling', which relies on genetic engineering of the cell-based therapeutic to ectopically express a so-called 'reporter gene' that enables contrast formation *in vivo* upon administration of a contrast agent (*e.g.* a reporter protein enabling the uptake or the binding of a contrast agent) 105 (Table 5 and Figure 2).

### *4.1 In vivo* imaging of directly labelled T-cell therapeutics

Requirements for T-cell labelling are dependent on the type of cell therapy. Tracers should be retained in or on the cells for the time required for their *in vivo* trafficking and proliferation without perturbing the function of these effector cells. The sensitivity of these imaging techniques is directly related to the specific activity reached and retention of radioactivity in the cells, and can be achieved by either passive membrane diffusion, binding membrane molecules or endocytosis. [^111^In]In-oxine and [^99m^Tc]Tc-HMPAO are routinely used for *ex vivo* direct cell labelling and tracking with SPECT for various inflammatory and infectious conditions 106, 107. Early clinical studies used *ex vivo* [^111^In]In-oxine labelling of CD4+ T-cells (1.2 x 10^9^ cells with 6.7 MBq) to demonstrate sequestration of CD4+ T-cells in Hodgkin's lymphoma lesions with SPECT imaging 108, or accumulation of adoptively transferred *ex vivo* expanded tumour-infiltrating lymphocytes in melanoma patients 109, 110. Eighteen patients were injected with 4.4-14 x 10^9^ cells with a specific activity of 55-255 kBq/10^6^ cells 110. More recently, this technique was used to assess the localisation of tumour-specific CTLs in the context of hemagglutinin-negative and hemagglutinin-expressing tumour models. [^111^In]In-oxine labelled CTLs specific for hemagglutinin redistributed to hemagglutinin-expressing tumours after 2h, where the signal continued to increase for up to 120h post-injection. In contrast to hemagglutinin-negative tumours, tumour-specific CTL infiltrated the core of hemagglutinin-positive tumours. Tumour antigen specificity was previously shown with intravital microscopy to be a requirement for tumour-infiltration of CTL cells 111. Currently, *ex vivo* [^111^In]In-oxine-labelled autologous CD8+ T-cells, isolated using CD8+ specific magnetic beads, are used to evaluate T-cell infiltration in early stage non-small cell lung cancer patients who receive anti-PD-L1 immunotherapy in neo-adjuvant setting (NCT03853187).

Particularly for clinical studies, the sensitivity of scintigraphy and SPECT is insufficient to track small numbers of cells in a large distribution volume 112. Moreover, the lack of direct quantification hampers its application in current immunotherapy. Clinical PET imaging is an attractive alternative for its higher sensitivity and direct quantification. Copper-64 offers a reasonable half-life (12h) and cell labelling strategies using lipophilic complexes such as PTSM 113-115, diethyldithiocarbamate 116 and tropolonate 117 have been utilised for PET imaging. However, rapid efflux (<50% retained after 5h) of these labelled complexes from cells and strong subsequent uptake in the liver restrict clinical use of copper-64 complexes. Longer half-life isotopes are also preferred for medium-to-long term tracking. For these reasons [^89^Zr]Zr-oxine (half-life 78.4h) labelling of cells for PET imaging has recently been introduced. Several immune cell types, including DCs, naïve and activated CTLs, and natural killer cells have been labelled efficiently with [^89^Zr]Zr-oxine, which enables their non-invasive *in vivo* tracking by PET/CT. Tumour targeting for seven days has been demonstrated using this methodology, with CTLs accumulating in the tumour and inducing tumour regression 118. Additionally, xenograft mouse models of glioblastoma and prostate tumours were studied with [^89^Zr]Zr-oxine labelled CAR-T 119, with CAR-T cells detectable for up to six days. [^89^Zr]Zr-oxine labelling of tumour cells was followed for 14 days in mice, with similar labelling efficiency as [^111^In]In-oxine. With better label retention of zirconium-89 (71-90%) after 24h compared to indium-111 (43-52%) and Good Manufacturing Practice (GMP)-compatible manufacturing protocols available, [^89^Zr]Zr-oxine is a potential candidate for future clinical cell labelling 120. More recently, [^89^Zr]Zr-oxine has been used to label γδT-cells for tracking in breast xenograft mouse model 121. The γδT-cells were found in tumours within 48h and presence was confirmed with histology.

Another method for direct-cell labelling is the use of chelators directly bound onto cell surface proteins instead of lipophilic agents like oxine, which circumvents the need for disruption of the plasma membrane during labelling 54. *In vivo* efflux from the radiolabelled cells and retention of free zirconium-89 in the bone marrow is of concern in clinical studies, as it would increase the effective dose for patients. Bansal *et al.* approached this issue using [^89^Zr]Zr-desferrioxamine-NCS ([^89^Zr]Zr-DBN) to label human mesenchymal stem cells 54. [^89^Zr]Zr-DBN conjugates to primary amines on proteins expressed on the cell surface and the label was retained on the cell surface for up to seven days without affecting cell viability, as determined by ApoTox-Glo viability, cytotoxicity, and caspase 3/7 apoptosis assay. *In vivo* distribution was followed to the myocardium, lung, liver, and bone, but no tumour models were studied. In another study, pegylated and ^64^Cu-labelled gold nanoparticles ([^64^Cu]Cu-Au-NP) were loaded into CAR-T by electroporation. *In vivo* localisation of the labelled CAR-T cells and free [^64^Cu]Cu-Au-NP was evaluated in two immunocompromised mice 122. Radioactive measurement of the organs showed that the ^64^Cu-labelled CAR-T cells were found primarily in the lungs, while the free [^64^Cu]Cu-Au-NP were observed in liver and spleen, illustrating that CAR T-cells can potentially be used as carriers for tumour delivery and to circumvent clearance through the liver and spleen. Theoretically, 'cell loading' depending on passive diffusion processes or 'cell binding' using surface proteins are inherently different methods. The efficiency of cell loading might depend on ratio of membrane surface area *versus* cell volume for example. Cell binding might be limited by the number of available amine-groups for chelation, number of receptor/targets present and their turnover. Furthermore, via these routes, radionuclides end up in different cellular compartments, which impact the radiation dose per organelle. To date, no comparative studies have been performed that investigate the potential consequences for *in vivo* imaging of *ex vivo* labelled T-cells.

To compensate for the poor spatial resolution of PET imaging and the longer, but nevertheless limited half-life of most radionuclides in relation to T-cell trafficking kinetics, methods to employ MR imaging for whole-body cell tracking have been pursued. Optimised highly derivatised cross-linked iron oxide nanoparticles (CLIO-HD) resulted in markedly efficient cell labelling, 300 µg Fe/mL/10^6^ cells, with no toxic effect, which allows semi-quantitative high-resolution whole-body MRI tracking in preclinical tumour models 123. *In vivo*, the imaging sensitivity was found to be 3 cells/voxel, at 8.5 T and an imaging time of 25 minutes, was achieved, and given its high spatial resolution extensive heterogenetic distribution of the recruited of T-cells at tumour site was observed.

Nanoparticles, as a cargo to deliver high payloads or multiple contrast agents have been employed to assess ACT 124. For example, the stable isotope fluorine-19 for cell labelling and tracking with MRI 125. Due to the negligible amounts of fluorine-19 in background tissue, no signal interference is present and therefore it should offer high sensitivity 126. Fluorine-19 MRI has also been used to image activated T cells *in vivo* over a 3-week period 127, whereas others using fluorinated nanoparticles in a diabetic murine model confirmed efficient *in vitro* labelling of autoreactive CD4+ and CD8+ T-cells but found limited utility for *in vivo* tracking 128. Although fluorine-19 MRI can quantify the amount of label at the site, the conversion of this value to cell numbers becomes less accurate over time, due to cell division and possible loss of label 129. Labels that are retained intracellularly for the apparent lifetime of the cell must be tested extensively for toxicity or other effects on cell function due to the presence of label 130. In addition, clearance of label from dead cells must also be studied 131, as some labels can persist after cell death, while others are cleared 132, even in the case of relatively similar fluorine-19 labels.

Direct labelling methods of cells have certain drawbacks in the form of radio- or chemical toxicity, which is related to physical properties of radionuclide (i.e. decay), carrier, exact intra-cellular localization and activity dose per cell. For example, oxines are lipophilic metal ion chelators that diffuse into the cell membrane and have been demonstrated to have cell toxicity over time 55. In addition, the indium-111 isotope emits Auger electrons with very short range, which could become radiotoxic in close proximity of the cell nucleus 133, which is a probably a general disadvantage of lipophilic chelators with release of free radiometals in the cytoplasm such as PTSM 92. In contrast, carriers that allow accumulation of radiometal in the endosomal compartment or outer cell membrane might result in less cell damage, but direct comparative studies are scarce. Recent publications include assays on cell function in relation to activity dose zirconium-89/cell, and demonstrated no negative effects when labelled <20 kBq/10^6^ cells 121, but others showed that up to 70-88 kBq/10^6^ cells was also tolerated without loss of viability or function 118, 119, for copper-64 specific activities of 32 kBq/10^6^ cells (*via* [^64^Cu]Cu-PTSM) negatively affected cell viability, but 9 kBq/10^6^ (*via* [^64^Cu]Cu-DOTA-KJ1-26 mAb-cOVA-TCR complex-labelling) was well-tolerated 92. Furthermore, leakage of radiotracers from cells is a major issue that needs consideration. Especially, long lived radioisotopes like zirconium-89 that accumulations in bone are able to increase radioactive dosing overtime 119. These potential negative effects on cell function might be considered less relevant when the labelled cells are terminally differentiated or mature effector cells with limited life span, which is the case for most current diagnostic applications, *e.g.* white blood cell scintigraphy in infectious diseases. However, toxicity renders these techniques less suitable for tracking therapeutic cells which are supposed to have long-lasting survival and replication *in vivo*. Repetitive imaging with short-lived radionuclides might be preferred for this reason, as will be discussed in the next paragraphs.

## *In vivo* imaging of T-cell therapeutics using reporter genes

Reporter gene imaging strategies are characterised by ectopic expression of a transporters or enzymes, which is passed on to and maintained in filial generations upon cell division, thereby enabling the assessment of *in vivo* localisation and survival through molecular imaging 134 (Table 5 and Figure 2). Notably, reporter gene methodology does not require complex *ex vivo* cell labelling facilities and is less prone to associated cell damage/toxicities. The Achilles heel of this type of indirect cell labelling is that it requires genetic engineering, which is currently more expensive, too. While this is neither a concern for preclinical experimentation nor for cell therapies already reliant on genetic engineering such as CAR‑T therapies 104, reporter gene-based cell tracking is also associated with a higher regulatory burden because of risks relating to aberrant viral integration. Gene-editing technologies such as CRISPR/Cas9 have been deployed to place insertions into so-called safe harbour locations (e.g. AAVS1, Rosa26 135), and thereby can reduce this risk.

A large variety of reporter genes have been described for *in vivo* cell tracking across several preclinical imaging technologies 136. While optical signal-generating protein reporters such as fluorescent and luminescent proteins have been very useful for preclinical research, they currently play no role in clinical cell tracking due to the intrinsic issues associated with deep imaging in humans and their foreign nature 136. For the purpose of *in vivo* tracking of cell-based immunotherapy in a clinical context, high sensitivity and reasonably high resolution paired with anatomical context information is desired. Radionuclide imaging (PET, SPECT) offers the best depth penetration and absolute quantification with preclinical resolutions ≤1 mm and clinical resolutions in the low millimetre range 137-139. Here, we limit the discussion to radionuclide reporters suitable for PET imaging as the latter is currently the most promising technology for clinical *in vivo* cell tracking given its high sensitivity 140-144 (Table 5 and Figure 2). Cell detection sensitivities are dependent on the reporter and its molecular imaging mechanism as well as the cellular levels of reporter expression. Cell detection sensitivities have been reported preclinically to be as good as tens of thousands cells for effector T-cells using various different reporter genes 142, and hundreds/thousands for larger cancer cells expressing the sodium iodide symporter (NIS) when detected using the PET radiotracer [^18^F]tetrafluoroborate ([^18^F]BF_4_^-^) 145.

Clinical feasibility of the adoptive cell therapy tracking concept was demonstrated recently in human glioblastoma patients. CTLs were engineered to express the viral reporter gene herpes simplex virus 1 thymidine kinase (HSV-1tk) alongside a glioblastoma-targeting interleukin-13 zetakine. PET imaging with the radiotracer 9-(4-[^18^F]fluoro-3-[hydroxymethyl]butyl)guanine ([^18^F]FHBG) before and after CTL administration alongside MRI imaging to provide anatomical context was performed in seven patients 146. Only small amounts of the radiotracer were taken up in glioblastomas prior to cell therapy administration, likely through the enhanced permeability and retention effect or blood brain barrier disruption. Significant uptake was found after administration of traceable CTLs, thereby clearly demonstrating that the engineered CTLs were indeed located in the glioblastomas. Limitations of the study were (i) the intracranial administration of the CTLs together with IL-2 (for CTL expansion), which might not be feasible routinely and for other cancers, and (ii) the choice of the reporter gene HSV-1*tk*, which is a foreign protein and has been found to be immunogenic 147.

Immunogenicity of reporter genes is a general concern for cell-based immunotherapies. The expression of a foreign reporter protein on the surface of an adoptively transferred cell therapy could lead to the recognition of the cell-based immunotherapy as foreign by the patient immune system, followed by destruction of this therapeutic by the patient immune system precluding the intended therapy from being efficacious. Consequently, endogenous human reporter proteins have been proposed and corresponding PET radiotracers have been developed (Table 5 and Figure 2). These reporters trade off immunogenicity against reduced contrast, which is caused by expression of the endogenous form in certain body locations. Hence, careful consideration during experimental design is required to choose the most suitable reporter gene for envisaged application. For example, the endogenous expression of NIS is restricted to thyroid, salivary and lachrymal glands, the stomach, and at lower levels to the testes and lactating mammary glands 148. A recent first-in-man study of NIS expression with its PET tracer [^18^F]BF_4_^-^ has demonstrated good contrast in other organs on the whole-body level 149. This showed that NIS is suitable for tracking adoptive cell therapies intended to target cancers in other organs than those with endogenous NIS expression. Moreover, NIS sensitively reports cell viability due to its dependence on the ATP-driven cellular Na^+^/K^+^ gradient 148, 150. A mechanistic difference as compared to the foreign HSV-1tk reporter gene is that NIS does not trap any of its radiotracer substrates when extra-thyroidally expressed. This can reduce the cellular doses per imaging session due to cellular radiotracer efflux. Conversely, loss of signal due to efflux during uptake may lower the sensitivity of this method to track small numbers of cells. NIS has been used to track cells on the preclinical level and has recently emerged for tracking CAR-T 143, 144, 151-159. Other notable human reporter genes with potential for cell tracking applications but requiring performance assessment in the context of adoptive T-cell tracking are listed in Table 5. Of those listed, the human somatostatin receptor subtype 2 (hSSTr2) stands out due to the existence of radiotracers with favourable renal excretion profiles that are already in clinical use.

In addition to contrast and molecular imaging mechanisms, it is also important to consider radiotracer excretion routes, radiotracer capacities to cross the blood-brain barrier (BBB) and radiotracer syntheses. For example, the corresponding short half-life radiotracers of human NIS, human thymidine 2 (hTK2; 160) and human cytidine kinases (hcDK; 141, 161) are excreted *via* the favourable renal excretion route. But importantly, none of them crosses the BBB. Furthermore, the complexity of radiotracer synthesis for hTK and hcDK is significantly higher as compared to the NIS PET radiotracer [^18^F]BF_4_^-^, for which also an automated synthesis protocol is available 162.

MRI provides excellent resolution, complementing PET imaging, and has the advantage of co-registration with soft-tissue anatomy and certain functional imaging parameters. MRI reporter genes have been developed over the past two decades, for example iron carrier proteins, transferrin, tyrosinase, β-galactosidase paired with activated contrast agents, or chemical exchange saturation transfer (CEST) reporters 163. However, standalone MRI imaging lacks sensitivity and consequently in the ability to detect MRI reporter gene-expressing cells at relevant concentrations.

In summary, PET reporter gene imaging of adoptive cell therapies is at the stage where a few options for clinical implementation are available for cell therapies that already require genetic engineering (*e.g.* CAR-T). *In vivo* imaging in these forthcoming studies will inform on *in vivo* localisation, possible mis-localisation and expansion within days/weeks of administration, and general cell therapy survival in individual patients. In contrast, adoptive cell therapies not requiring genetic engineering for efficacy (*e.g.* TIL, γδT) are unlikely to be assessed in this manner as the regulatory burden added by genetic engineering for the sole purpose of cell tracking is too high. However, for the latter suitable *in vivo* imaging approaches, at least to assess initial *in vivo* distribution, are available through various direct cell-labelling methodologies, discussed in previous paragraphs.

## Discussion and future perspectives

In the quest to control cancer we now face the challenge to translate our increasing insights in the cancer-immunity cycle into more effective immune therapies. Similar to the instrumental role of *in vivo* imaging in understanding the complex interactions of cancer and the immune system in preclinical models, there lies a great task for imaging immune responses in patients to realise the potential of immunotherapy in the clinic. In this review, we focussed on T-cell responses under several immune therapeutic strategies, as 'T-cells are the drug' in both immune checkpoint inhibition and cell-based therapies.

At present, clinical imaging tracers for immune-oncology are scarce, and the introduction of new imaging tracers for clinical use falls behind the speed of immunotherapy development 13, 14. At least two obstacles underlying this observation can be noted. First, regulatory hurdles and lack of funding to develop a full GMP-grade product dossier for FDA and EMA approval impede the translation of novel tracers 164-167. Second, both regulatory bodies and oncological community still adhere to anatomical imaging biomarkers such as CT to evaluate tumour size as surrogate endpoint for efficacy. Novel drugs are approved based on RECIST criteria, although immune related criteria (iRECIST, 168) are available and roadmaps for imaging biomarker development have been proposed 169.

Underlying these practical issues is perhaps the paucity of collaborations between physician and laboratory in the academia, pharmaceutical industries, scientific communities and regulatory bodies to discuss the potential roles of molecular imaging in immune therapy. Each of these partners has complementary contributions in achieving the shared goal of effective immune therapy; academic labs can offer high level expertise on specific topics, and physicians are most aware of the needs and issues from a patients' perspective, which should fuel the directions of research. On the other hand, pharmaceutical industry partners have excellent research and development departments, specialized in discovery of targets and GMP-production of targeting agents; plus, they control the infrastructure necessary to reach out to multi-center clinical studies. Lastly, scientific communities communicate with regulatory bodies via the approval of guidelines, harmonization protocols and recommendations, which enable the implementation of imaging techniques in routine practice. In such collaborative effort, identifying a robust biological endpoint and addressing relevant clinical questions should catalyse the translation from a preclinical imaging agent to a clinically utile protocol. This review was written to serve this process in particular; *in vivo* imaging should provide tools for 1) more efficient immunotherapy development, 2) individualised treatment planning and 3) guide the implementation of cell-based therapies (Figure 3).

As a tool for more efficient immunotherapy development, imaging should address for example *in vivo* distribution and re-distribution of monoclonal antibodies and cell-based therapy by direct labelling, and target well-established endpoints related to the mechanism of action of these immunotherapies. Thus, addressing critical requirements for effective immunotherapy; does the drug reach its target, does it induce the supposed effects *in vivo*, is there a dose-effect relation? Following recent durable responses achieved in the clinic with immune-modulating therapies, the number of immune-oncological drug combinations exploded 13, 14, mostly empirically designed or based on mouse models. Despite these admirable endeavours, we should provide a word of caution as mouse models are only moderately representative of the complex human immune system and tumour microenvironment in terms of cellular composition and diversity. Moreover, mouse models often comprise of small tumours with aberrant vascularisation when implanted subcutaneously, factors that cannot replicate the bulky tumours that arise from parenchymal tissues in patients. These tumours have highly heterogeneous perfusion and necrotic regions that are not accessible for large proteins such as antibodies.

In this respect, labelling the therapeutic agent itself to assess *in vivo* biodistribution and in particular the achieved local tumour doses, has revealed striking intra- and inter-metastatic heterogeneity and confirmed that patients with higher target presence and availability, *e.g.* PD-1/PD-L1 expression, have increased tendency to respond. The encouraging results of the pioneering clinical studies exploiting radiolabelled nivolumab and atezolizumab have laid the format for several on-going studies using radiolabelled antibodies targeting immune checkpoints 96, 170. Previous analysis of PET imaging of radiolabelled therapeutic antibodies have demonstrated the cost-effectiveness of such approach 171, refuting the arguments of high costs per imaging procedure. Initiated by these recent clinical studies on [^89^Zr]-labelled antibodies and urged by undesired adverse events in CAR T-cell therapy 96, 103, 104, 170, these issues require whole body, quantitative imaging techniques using long-lived tracers such as PET to assess a second *in vivo* biodistribution following days upon first administration. Given the requisite of high affinity, high specificity and high sensitivity, other scaffolds targeting T-cell populations for PET imaging are currently under development in different disease types that might also be relevant to immunotherapy 172, 173.

Current tools for translational research aiming to increase our understanding of factors underlying success or failure of immunotherapy, *e.g.* tumour biopsies and blood-based assays, provide snapshots of these interactions from single body compartment and which require complex analyses pipelines to link fragments of information to composite endpoints such as tumour response or survival. Imaging tools that accurately reflect the presence of specific immune cell populations and their effector functions, targeted by immunotherapy, in a dynamic fashion provide a surrogate endpoint that can be evaluated real-time and in conjunction to other biomarkers. In the near future, tools to assess T-cell effector functions related to clinically-meaningful surrogate endpoints, such as granzyme B expression or IFNγ presence will become available 99, 100, fostering the use of imaging to not only evaluate the presence of immune cell populations, but also their actual *in vivo* function. During the translational process, validation of imaging findings on human tissue is essential, but mostly not feasible in clinical studies for medical-ethical reasons. This is an aspect slowing the optimisation and selection of promising candidate tracers. Again, the requirement for whole body, quantitative evaluation favours PET as imaging modality of choice.

A complementary strategy to increase the efficacy of immunotherapy is to improve the selection of patients who are likely to respond. The dynamic and quantitative assessment of presence of drug target on whole body scale therefore is highly desired. Peptide-based or antibody fragment-based approaches outperform radiolabelled antibodies in this respect 89, 174-176, as their fast kinetics and less dependency on tissue structure and perfusion better match this goal. In the future, quantitative assessment of induction of immune activation during immunotherapy, *e.g.* by measuring expansion of T-cell populations, T-cell specific activation markers or effector functions, would allow early adaptation of treatment to individual patient characteristics. In contrast to current radiological evaluation of tumours size at late time points and with poor correlation to mechanism of action of immune therapeutic drugs, it is tempting to speculate that certain tracers would allow tailoring treatment based on early surrogate endpoints. Imaging OX40 expression provides a glimpse in this direction 70, as is granzyme B or IFNγ imaging, but all are far from validated thresholds that can be relied on for clinical decision-making.

Lastly, tracers for long-term imaging without perturbing functionality of therapeutic cells are a prerequisite to guide the full implementation of cell-based therapy, and will need to rely on genetically encoded reporters. Imaging the dynamics and function of adoptively transferred T-cells is critical to understand the methods that are employed by T-cells to interact with tumour cells. The most prominent example is graft-*versus*-host disease; current diagnosis is made upon clinical symptoms indicating organ injury using invasive techniques such as tissue biopsies. Non-invasive imaging of early stages in immune activation could potentially be implemented for early diagnosis and thus preventing additional procedure-related organ injury 75.

PET imaging in this respect has most of the desired features (sensitive, availability, easy quantification) but it involves radiation, lacks spatial resolution, and has no possibility to measure multiple labels simultaneously in clinical setting. A multimodal imaging approach, combining PET with diagnostic CT or MR provides a readily available solution to the poor spatial resolution. In this respect, nanoparticles are of interest for emerging multimodal imaging techniques, providing carriers with increased capacity for loading (multiple) contrast agents that can be modified to enhance cellular uptake, targeting to specific immune cell populations or prolonged intracellular retention. Other approaches are to reduce noise in ^89^Zr-labelled antibody images, which have shown to have good repeatability coefficient (<6%) in manually delineated organs, likely independent of the antibody specificity as several antibodies had been used with similar results. Noise-induced variability in bone marrow and blood pool was higher, due to lower activity 177. Furthermore, technological developments on the hardware of PET scanners, such as total-body PET, resulting in unprecedented sensitivity allows to reduce the injected dose to a minimum and in the same time perform dynamic imaging at high temporal resolution 178.

The commonly used radionuclides fluorine-18 and zirconium-89 both have their strengths; fluorine-18 with 109 minutes half-life reduces effective dose and allows rapid succession of imaging procedures. On the downside, fluorine-18 requires on-site production, which consequently means harmonization of production and acquisition protocols if multi-centre studies are planned. On the other hand, zirconium-89 with 78h half-life allows tracking over several days following a single injection and allows shipment to other institutes, reduces the number of production sites, convenient for harmonization thus favourable logistics and infrastructure facilitating multi-centre studies 179.

In summary, there is a clear need for means to interrogate T-cell responses during immunotherapy. This is in line with increasing pressure for better tools to assess the success of cell therapies at least at early stages but ideally in the long-term, both from a regulatory and reimbursement standpoint. This review provides arguments to match promising imaging tools with relevant clinical needs, aiming to foster implementation of clinical *in vivo* imaging to further the development and translation of anti-cancer immunotherapy.

## Figures and Tables

**Figure 9:**
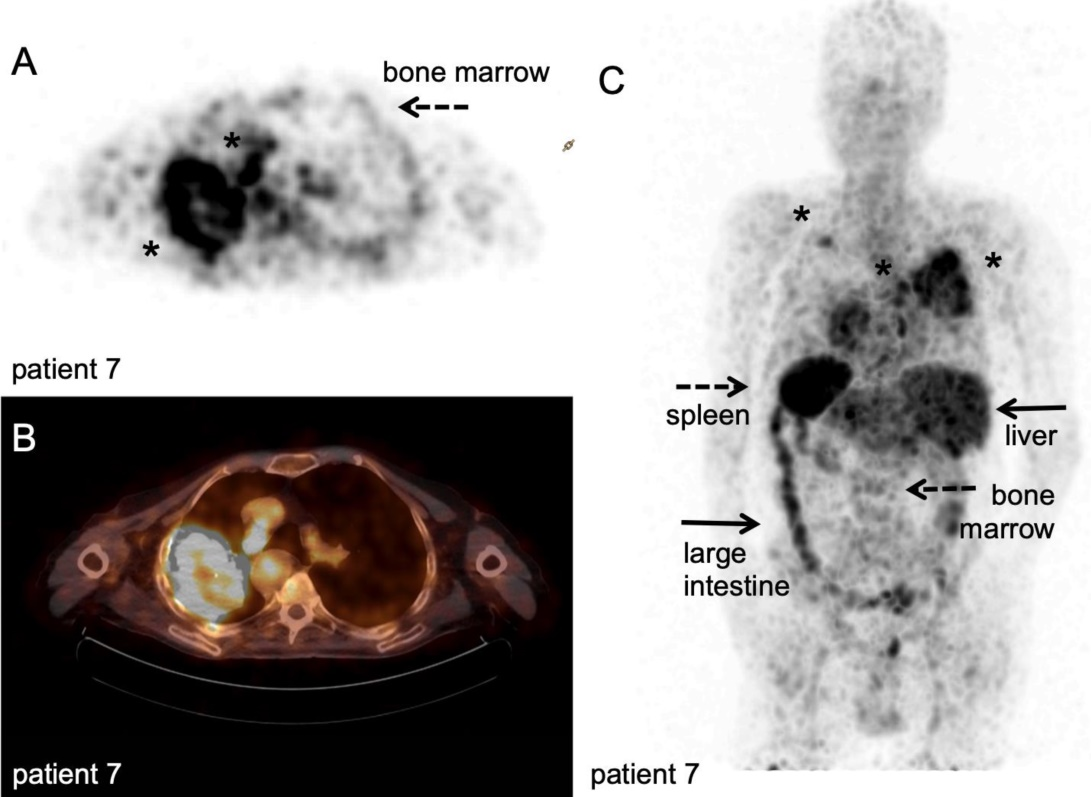
**^89^Zr-nivolumab PET/CT scan.** Metastatic non-small cell lung cancer patient, scanned at 162 hours after IV injection of 37 MBq [^89^Zr]Zr-DFO-nivolumab. A-B: transversal PET and fused PET/CT images; a large primary lung tumour in the right upper lobe and medial to the large mass a second lesion (asterisk). C: Maximum intensity project, posterior view; note the high uptake in the spleen (dashed arrow) and liver (filled arrows), as well as uptake in the bone marrow compartment (dashed arrow) and large intestine (filled arrow). Both lesions in the right lung were visualised (asterisk), as well as another lesion in the left upper lobe (asterisk). *With courtesy of J. de Lange*

**Table 1:** T-cell surface markers used for *in vivo* imaging.

Name/Target	Type	Tracer / substrate	Discussion	Active clinical trials	Ref
murine OX40 receptor (CD134) (murine target)	Antibody	[^64^Cu]Cu-DOTA-AbOX40	- Expression per cell is dynamic and might hamper correlation with cell numbers	NCT02318394	70
human Interleukin-2 receptor alpha chain (CD25) (murine and human target)	Cytokine	[^99m^Tc]Tc-HYNIC-IL-2	- Expression per cell is dynamic - IL-2 has short plasma half-life 7-14 minutes - Radiolabelled IL-2 is biologically active	n/a	65, 180
		[^18^F]FB-IL-2		NCT02922283	66
murine CD3 (murine target)	Antibody	[^89^Zr]Zr-p-isothiocyanatobenzyldeferoxamine-CD3	- Receptor present on both CD8 and CD4 T-cells - Probe dilution due to cell division	n/a	86
murine CD4 (murine target)	Cys-diabody	[^89^Zr]Zr-malDFO-GK1.5 cDb	- Rapid clearance as compared to full antibodies - Less dependent on tissue structure and perfusion	n/a	90
murine CD8 (murine target)	Cys-diabody	[^89^Zr]Zr-malDFO-169 cDb		n/a	89
human CD8 (human target)	Minibody	[^89^Zr]Zr-Df-IAB22M2C		NCT03802123, NCT03610061	181
murine T-cell receptor (murine target)	Antibody	[^64^Cu]Cu-cOVA-TCR	- Internalization of TCR-complex results in higher specific activity - Binding of the TCR might induce signalling - Probe dilution due to cell division	n/a	92
murine T-cell receptor beta domain (murine target)	F(ab')2 fragment	[^89^Zr]Zr-Df-aTCRmu-F(ab')2		n/a	93

**Table 2:** Metabolic pathways for T-cell imaging.

Name/Target	Type	Tracer / substrate	Discussion	Active clinical trial	Ref
DNA synthesis	Deoxyribonucleotide	1-(2′-deoxy-2′-[^18^F]fluoroarabinofuranosyl) cytosine	- Rapid probe catabolism by cytidine deaminase in humans - In general lower uptake in tumours as compared to [^18^F]FDG.	n/a	71, 72
		2'-deoxy-2'-[^18^F]fluoro-9-β-⫐-arabinofuranosylguanine	- Predominantly *via* dGK	NCT03311672, NCT03142204, NCT03007719	75
		3'-deoxy-3'-[^18^F]fluorothymidine	- In general lower uptake in tumours as compared to [^18^F]FDG.	n/a	78, 79, 182
		2-chloro-2′-deoxy-2′-[18F]fluoro-9-β-D-arabinofuranosyl-adenine		NCT03409419	61, 62
Amino acid metabolism	Amino acid L-leucine analogue	trans-1'-amino-3'-[^18^F]fluorocyclobutanecarboxylic acid	- Rapid biological clearance in all tissue.	n/a	76, 77, 183
Glycolysis	Glucose analogue	2'-deoxy-2'-[^18^F]fluoro-⫐-glucose	- (Very) low specificity for T-cells - No quantitative correlation with T-cell activation has been observed	used in numerous trials for tumor response evaluation	80-84

**Table 3:** Imaging targets related to T-cell effector function.

Name/Target	Type	Tracer / substrate	Discussion	Active clinical trials	Ref
human PD-1 (murine and human target)	Antibody	[^64^Cu]Cu-DOTA-PD-1	- Slow accumulation in peripheral tissue, thus multiple day-acquisition protocols - No established correlation with molecule expression levels - Accurate reflection of *in vivo* biodistribution of the therapeutic antibody	n/a	95, 96
		[^89^Zr]Zr-nivolumab		n/a	
		[^89^Zr]Zr-pembrolizumab		NCT03065764, NCT02760225	
human CTLA-4 (murine target)	Antibody	[^64^Cu]Cu-DOTA-anti-CTLA-4;	.- Accurate reflection of *in vivo* biodistribution of the therapeutic antibody	n/a	97
		[^89^Zr]Zr-ipilimumab		NCT03313323	
Granzyme B (murine target)	Peptide	[^68^Ga]Ga-NOTA-GZP	- Less dependent on tissue perfusion and perhaps better reflection of actual molecule expression levels	n/a	98
Interferon gamma (murine target)	Antibody	[^89^Zr]Zr-anti-IFNγ		n/a	100

**Table 4:** *Ex vivo* direct T-cell labelling techniques.

Name/Target	Type	Tracer / substrate	Discussion	Active clinical trials	Ref
Passive membrane diffusion (murine and human target)	Chelating agent	[^111^In]In-oxine; [^89^Zr]Zr-oxine	- Concerns of impaired viability/functionality of T-cells - Efflux from the cell	NCT03853187	21, 106, 108-111, 118-121
Passive membrane diffusion (human target)	Chelating agent	[^99m^Tc]Tc-Hexamethyl-propyleneamine oxime (HMPAO)		n/a	107
Passive membrane diffusion (murine target)	Carrier molecule, chelating agent	[^64^Cu]Cu- Pyruvaldehyde-bis(N^4^-methylthiosemicarbazone) (PTSM)	Rapid efflux of labelled from cells. Cell toxicity without PEGylation of PEI.	n/a	113-117
		[^64^Cu]Cu-Diethylthiocarbamate (PEI)			
		[^64^Cu]Cu-tropolonate			
Cell surface bound peptides (murine target)	Chelating agent	[^89^Zr]Zr- p-Isothiocyanatobenzyl-desferrioxamine (DBN)	Probe dilution due to cell division. Radiotoxicity of bone when chelator is release from cell surface.		54
Endocytosis/phagocytosis (murine target)	Nanoparticles	[^64^Cu]Cu-Au-NP	Electroporation used for T-cell labelling		122
Endocytosis/phagocytosis (murine target)	Nanoparticles	Highly derivatised crosslinked iron oxide nanoparticle (CLIO-HD)	*Ex vivo* labelling of cells. Toxicity at high intracellular concentrations.		123
Endocytosis/phagocytosis (murine target)	Stable isotope	^19^F-NP	Probe dilution due to cell division. Probe persistence after cell death.		128

**Table 5:** Promising PET reporter gene strategies.

Name	Type	Properties	Tracer / substrate	Excretion	Limitations	Ref
Somatostatin receptor type 2 (SSTr2)	Cell surface receptor	G protein-coupled receptor (GPCR).	[^68^Ga]Ga‑DOTATOC, [^68^Ga]Ga‑DOTATATE.^&^	Renal	Endogenous expression in brain, adrenal glands, kidneys, spleen, stomach and many tumours (*i.e.* SCLC, pituitary, endocrine, pancreatic, paraganglioma, medullary thyroid carcinoma, pheochromocytoma); tracers may cause cell signalling and change proliferation.	184-187
Dopamin receptor (D_2_R)	Cell surface receptor	GPCR; tracers cross BBB.	[^18^F]FESP, [^11^C]C-Raclopride, [^11^C]C-N-methylspiperone.	Renal and hepatobiliary	Slow clearance of [^18^F]FESP; high background in the pituitary gland and striatum due to endogenous expression.	188-191
Sodium iodide symporter (NIS)	Transporter	Symports sodium ions.	[^124^I]I^-^, [^18^F]BF_4_^-^, [^18^F]SO_3_F^-^, [^18^F]PF_6_^-^.^&^	Renal	NIS is endogenously expressed in thyroid, stomach, lacrimal, salivary and lactating mammary glands, small intestine, choroid plexus, testicles; tracers do not cross BBB.	192-196
Dopamin transporter (DAT)	Transporter	NaCl-dependent; tracers cross BBB.	[^11^C]CFT, [^11^C]C-PE2I, [^18^F]FP-CIT.^&^	Renal and hepatobiliary	Data about DAT use as reporter gene are scarce while tracers are widely used.	Patent by 197
Pyruvate kinase M2	Enzyme	Expression during development; in cancers. Tracer crosses BBB.	[^18^F]F-DASA-23	Renal and hepatobiliary	Background in organs of excretion route.	198
Human thymidine kinase (hmtk2/hΔTK2)	Enzyme	Kinase causing cellular tracer trapping.	[^124^I]I-FIAU**, [^18^F]FEAU, [^18^F]FMAU (hTK2-N93D/L109F).	Renal	Tracers do not cross the BBB; Endogenous signals in gall bladder, intestine and organs involved in clearance.	160
Deoxycytidine kinase (hdCK)	Enzyme	Kinase causing cellular tracer trapping.	[^124^I]I-FIAU**, [^18^F]FEAU.	Renal	Tracers do not cross the BBB; Endogenous signals in gall bladder, intestine and organs involved in clearance.	141, 161
Glutamate carboxypeptidase 2 (PSMA)	Cell surface enzyme	High expression in prostate.	[^18^F]F-DCFPyL, [^18^F]F-DCFBC, [^68^Ga]Ga-PSMA-11.^&^	Renal	Background in organs of excretion route; tracers do not cross BBB.	199
Estrogen receptor α ligand binding domain	Artificial cell surface molecule	No physiological function reported; tracer crosses BBB.	[^18^F]FES.	Renal and hepatobiliary		200

## Abbreviations

ACT: adoptive cell transfer
APC: antigen presenting cells
BBB: blood-brain barrier
CAR T-cells: chimeric antigen receptor T-cells
CEST: chemical exchange saturation transfer
CTL: cytotoxic T lymphocyte
CTLA-4: cytotoxic T lymphocyte-associated antigen 4
DC: dendritic cell
EMA: European Medicines Agency
FDA: Food and Drug Administration
GMP: Good Manufacturing Practice
GVHD: acute graft-*versus*-host-disease
hcDK: human cytidine kinases
hSSTr2: human somatostatin receptor subtype 2
hTK2: human thymidine 2
HSV-1tk: herpes simplex virus 1 thymidine kinase
INFγ: interferon gamma
LN: lymph node
MR: magnetic resonance
MRI: magnetic resonance imaging
NIS: sodium iodide symporter
PET: positron emission tomography
PD-1: programmed cell death receptor 1
PD-L1: programmed cell death ligand 1
SLO: secondary lymphoid organs
SPECT: single photon emission computed tomography
TCR: T-cell receptor
TIL: tumour infiltrating lymphocytes
